# Degradation of plastics and plastic-degrading bacteria in cold marine habitats

**DOI:** 10.1007/s00253-018-9195-y

**Published:** 2018-07-11

**Authors:** Aneta K. Urbanek, Waldemar Rymowicz, Aleksandra M. Mirończuk

**Affiliations:** Department of Biotechnology and Food Microbiology, Wroclaw University of Environmental and Life Sciences, Chełmońskiego 37, 51-630 Wrocław, Poland

**Keywords:** Plastic wastes, Biofilm, Microorganisms, Cold marine environment, Biodegradation

## Abstract

Synthetic plastics present in everyday materials constitute the main anthropogenic debris entering the Earth’s oceans. The oceans provide important and valuable resources such as food, energy, and water. They are also the main way of international trade and the main stabilizer of the climate. Hence, changes in the marine ecosystem caused by anthropogenic influences such as plastic pollution can have a dramatic impact on a global scale. Although the problem of plastics still remains unsolved, different ways are being considered to reduce their impact on the environment. One of them is to use microorganisms capable of degradation of plastic. A particularly interesting area is the application of microorganisms isolated from cold regions in view of their unique characteristics. Nevertheless, the interactions between plastic and microorganisms are still poorly known. Here, we present a review of current knowledge on plastic degradation and plastic-microorganism interactions in cold marine habitats. Moreover, we highlight the advantages of microorganisms isolated from this environment for eliminating plastic waste from ecosystems.

## Introduction

**Fig. 1:**
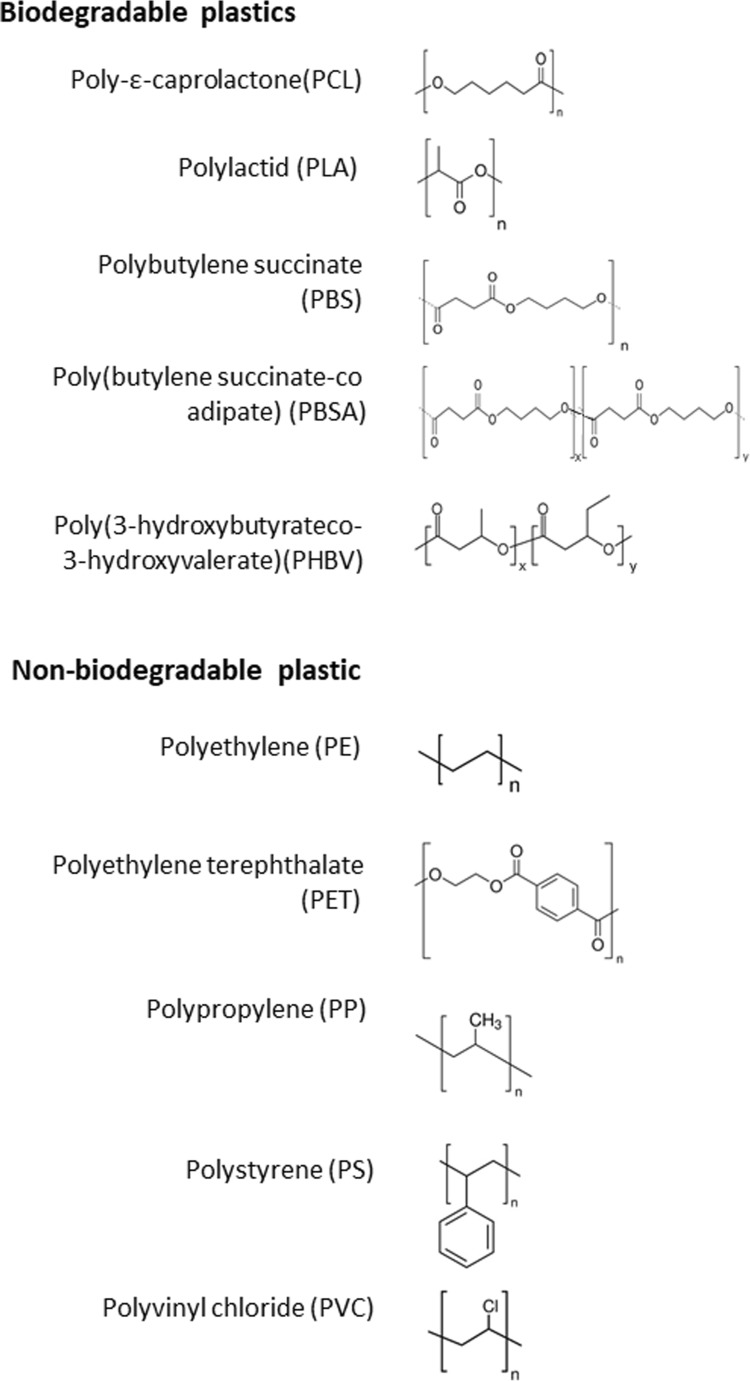
Structures of the common plastics

Synthetic plastic production is one of the fastest growing fields of global industry. Despite the fact that plastics have been used in daily life for 100 years, the beginning of large-scale production dates back to 1950 (Geyer et al. 2017). The numerous properties that make plastics superior to other materials in many applications have led to a 20-fold increase in the scale of plastic production over the five decades since 1964 (Ellen MacArthur Foundation 2016), exceeding 300 million tons per year (PlasticsEurope 2015) and reaching 335 million tons in 2015 (PlasticsEurope 2017). Furthermore, it is foreseen that production of plastics will double over the next 20 years and almost quadruple by 2050 (Ellen MacArthur Foundation 2016). About 80% of the total global plastic usage constitutes petrochemical plastic, such as polyvinyl chloride (PVC), polyethylene (PE), polypropylene (PP), polystyrene (PS), and polyethylene terephthalate (PET) (Fig. 1). Although plastic materials constitute an integral part of the global economy, the issues associated with their extensive application cannot be ignored. Accumulation of plastic litter occurs in the marine environment mostly, where it is hard to find any area that is unaffected by human influence (Halpern et al. 2008). Worldwide accumulation of plastic on the surface of the open ocean is frequently found in the convergence zones of each of the five subtropical gyres (Cózar et al. 2014). However, plastic debris has been found in high concentrations (hundreds of thousands of pieces per square kilometer) of the Greenland and Barents seas (Cózar et al. 2017). Also, in the Antarctic marine system (Southern Ocean), plastic debris has been found on the surface and in deep-sea sediments. In these regions, mainly microplastics (< 5 mm) and mesoplastics (< 5 cm) have been found (Waller et al. 2017). It was estimated that every year, 10 to 20 million tons of plastics leak into the oceans (UNEP 2014). Since 2015, approximately 6300 million tons of plastic waste have been generated (Geyer et al. 2017), of which a significant percentage has found its way to the environment as a result of uncontrolled dumping of wastes. The main limitation of conventional petroleum-based plastics is the fact that they fragmented under abiotic factors (UV radiation, temperature, physical stress) in a long time, and they cannot be completely decomposed and assimilated by microorganisms (biotic factors) in a biodegradation process. Crucial characteristics responsible for plastics’ resistance to biodegradation include a long-chain polymer structure, a high molecular weight (MW), lack of a favorable functional group, hydrophobicity, and crystallinity (Wilkes and Aristilde 2017). A high MW is a crucial obstacle, because large compounds cannot be transported across the cellular membrane of microorganisms. Thus, long-chain polymers have to be first depolymerized to smaller monomers before they can cross the cell membrane (Shah et al., 2008). Next, monomers can pass through the cell membrane, followed by assimilation by intracellular metabolism (Kolvenbach et al. 2014). Due to the fact that most petrochemical plastics are not biodegradable, new biodegradable plastics (BPs) have been developed and some of them have already been introduced to the market. Nowadays, there are many products available (bottles, packages) that are made from biodegradable plastics such as poly(lactic acid) (PLA), poly(ε-caprolactone) (PCL), poly(butylene succinate) (PBS), or poly(butylene succinate-co-butylene adipate) (PBSA) (Fig. 1). Biodegradable plastics, which may be classified as being either bio-based or petrochemical-based (Song et al. 2009), can be degraded in an eco-friendly way by microorganisms, resulting in the fragmentation of material via microbial enzymatic activities and bond cleavage (Pathak and Navneet 2017).

Plastic wastes might be dangerous for the natural environment due to accumulation in the rivers and oceans, where the formation of plastic islands (e.g., the Great Pacific Garbage Patch) is confirmed (Eriksen et al. 2014, Lebreton et al., 2018). Moreover, as the result of abiotic degradation of conventional plastic caused by UV radiation, oxygen, temperature, and physical stress (Geweret et al. 2015), slowly degrading large plastic items generate microplastic particles which can spread over long distances by wind-driven ocean surface layer circulation (Thevenon et al. 2014). Thus, places located far away from pollution sources are affected by plastic wastes. There is also a concern that plastic is a source of toxic chemicals such as polychlorinated biphenyls or phthalates and bisphenol A (Bryant et al. 2016). These contaminations have also a significant influence on marine fauna due to entanglement, suffocation, and disruption of digestion in birds, fish, mammals, and turtles (Derraik 2002). According to research of the Alfred Wegener Institute, Helmholtz Centre for Polar and Marine Research, 1506 species are affected by the litter (Tekman et al. 2017).

In this study, we focus on the problem of plastic pollution in cold regions, especially in the marine environment. It is necessary to understand that oceans not only accumulate plastics at certain points but also transfer them to distant virgin regions such as the Arctic and Antarctic. Moreover, we try to explain the interaction between marine microorganisms and plastic waste drifting in the ocean. Based on the current knowledge, we have gathered information about plastic-degrading bacteria in cold marine habitats and explain the advantages of searching for them.

## Plastic waste in the cold marine environment

**Fig. 2:**
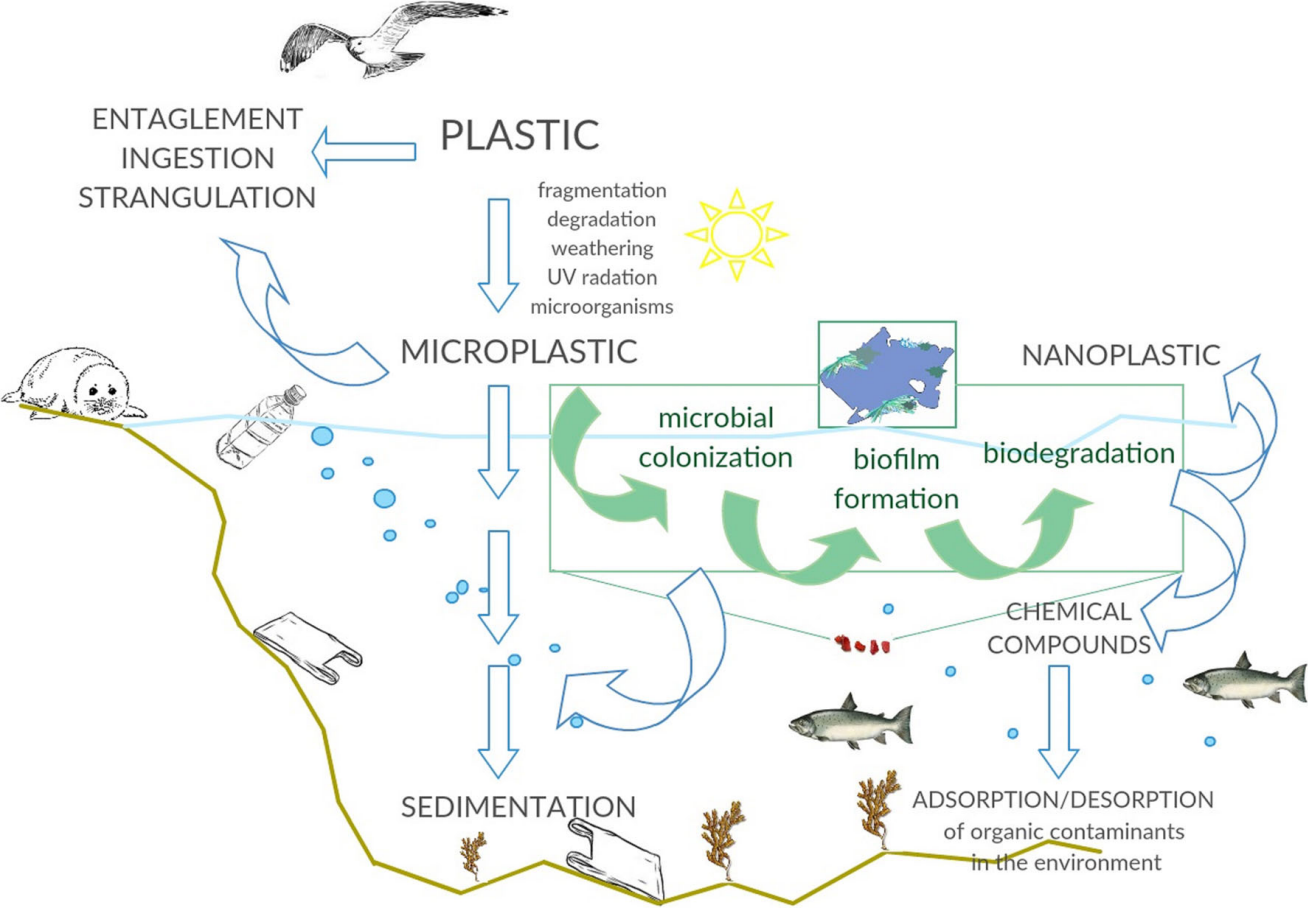
Potential interactions between marine microorganisms and microplastics in marine environment

The main sources of synthetic plastic waste in the marine environment are waste from coastal tourism, fishing, marine industries, and manufacturing of plastic products which have a direct impact on seas and oceans (Cole et al. 2011; Veiga et al. 2016). Furthermore, the indirect path of plastic dispersion into the marine environment is also significant. Pollutants from the cosmetic industry or households firstly enter the rivers and drainage systems and then reach the seas and oceans (Cole et al. 2011), which might be related to the higher concentration of plastic near the coasts and river estuaries (Maes et al. 2017). It should be noted that plastic fragments smaller than 5 mm in diameter that directly enter the environment (e.g., from facial cleansers and cosmetics) are described as primary microplastics, whereas particles formed as a consequence of fragmentation of larger items are called secondary microplastics (Veiga et al. 2016). Slow degradation of large plastic fragments and generation of microplastic is caused by UV radiation and mechanical forces and is a facilitating factor in the transfer of plastic over long distances (Thevenon et al. 2014). Depending on its density, plastic accumulates in the water column of central convergence zones and floats at the surface (Cózar et al. 2014; Pauli et al. 2017) or sinks to the sea floor after loading with biotic and abiotic dissolved compounds (Bergmann and Klages 2012; Derraik 2002). At the same time, there occurs settlement of the biomass on floating plastic, which is called biofouling (van Sebille et al. 2016) (Fig. 2). Marine biofouling refers to the colonization of man-made products (including plastics) submerged in seawater by biotic and abiotic factors, microorganisms, plants, and animals. Biofouling involves five main phases: adsorption, immobilization, consolidation, microfouling, and macrofouling. Bacteria are crucial for biofouling since they take part in primary colonization of the surfaces during primary microfouling (Selim et al., 2017). Microfouling undergoes two steps, primary (primary colonizers, bacteria and diatoms) and secondary colonization (Selim et al., 2017). Due to the different density of particles and possibility of transportation, plastic wastes could be gathered from highly populated latitudes, leading to accumulation in the cold seas and polar regions (Waller et al. 2017). The first report about plastic pollution in offshore basins of the North Atlantic Ocean was published in the 1970s. At that time, attention was paid to the concentration and characteristics of plastic, which reached 3500 pieces per km^2^. The particles in pellet shape and diameter not exceeding 5 mm were attached by diatoms and hydroids (Carpenter and Smith 1972). One of the latest studies presenting the range of microplastic contamination was conducted in sediments of the Southern North Sea and at the sea surface of North West Europe. The floating concentration reached 0–1.5 microplastic particles/m^3^, while microplastic concentrations in sediments varied in the range 0–3146 particles/kg of dry weight sediment (Maes et al. 2017). This observation could support the theory that the litter in sediment can persist for a long time, as degradation rates may be lower due to low ambient temperature (0–4 °C), low energy input, and the absence of sunlight (Bergmann and Klages 2012), in comparison to plastics present in surface waters, which are more prone to degradation (Caruso 2015). In particular, the reduction in UV light delivery might have the greatest impact due to the key role in initiation of the oxidative process (O’Brine and Thompson 2010). Moreover, the level of oxygen in deep-sea sediments can be rather low or can be completely anoxic. Oxidative catabolic activities that would be necessary for degradation of hydrocarbon polymers therefore are unlikely to occur under these conditions. Interestingly, another study shows that 2 years are needed for plastic wastes released from the UK to reach the Barents Sea and the Arctic (van Sebille et al. 2016). It should be noted that in contrast to large plastic particles affecting fish and birds, depending on concentration, pieces of microplastic can represent a threat to organisms at lower trophic levels (Rummel et al. 2017) such as zooplankton and mussels (Caruso 2015). Floating microplastics (< 1 mm) can be easily ingested by zooplankton and consequently are egested with their fecal pellets. These pellets are a source of food for marine organisms, constituting a vector for faster vertical transport (Cole et al., 2016).

## Interaction between microorganisms and plastic in cold marine habitats

Microorganisms are able to survive under various conditions, and many cold places such as permanently ice-covered lakes, sea ice, snow, permafrost soils, cloud droplets, rock environment, or glacial ice have been found to be habitants for bacteria (Cameron et al. 2012; Junge et al. 2004; Russell 1990; Yadav et al. 2017). The abundance of microorganisms in marine ecosystems reaches up to hundreds of millions of bacterial cells in a gram of wet marine sediment (Harrison et al. 2011). Furthermore, it is assumed that any surface in the marine environment is colonized with macro- and microorganisms (Eich et al. 2015; Lobelle and Cunliffe 2011). Therefore, sunken or floating plastic wastes are not free of the influence of microorganisms. Despite the fact that microorganisms can colonize all plastic that is introduced to the marine environment (De Tender et al. 2015; Eich et al. 2015; Pauli et al. 2017; Rummel et al. 2017), a limited number of studies have been conducted on the interactions between plastic and marine microbiota (Bryant et al. 2016; Harrison et al. 2011). Unfortunately, the precise mechanisms of the bacterial attachment on the plastic surface are poorly known. Attachment to surfaces and biofilm development are a well-known strategy of bacteria for surviving a variety of conditions in the marine environment (Junge et al. 2004), including the ability to form stable consortia, horizontal gene exchange, accumulation of nutrients, and protection against toxic substances (Rummel et al. 2017). In seawater, bacterial colonization on plastic material starts almost immediately. Within a few hours, microorganisms are able to form microbial assemblages and cover the surface of plastic, which is defined as attachment. During this stage, microbial assemblages might catalyze metabolic reactions that lead to the adsorption, desorption, and fragmentation of microplastic-associated compounds or even the breakdown of the debris itself (Harrison et al. 2011). Building a biofilm provides a proper platform for the settlement of other organisms (Pauli et al. 2017) such as microalgae (including diatoms, flagellates, protists) and microscopic fungi. The abundance ratio between these organisms can vary; the cell ratio of bacteria/diatoms/flagellates on polymer plates from the White Sea was 640:4:1, whereas the proportion of other organisms was about 0.15% (Salta et al. 2013). Due to phylogenetic, functional, and ecological variety, biofilms are termed a microbial assemblage, biofouling community, or periphyton (Rummel et al. 2017), and moreover, due to being distinct from the surrounding water, they are also called the “plastisphere” (Zettler et al. 2013). Biofouling increases the density of the particle, and thus, it may sink to the seafloor (Pauli et al. 2017). It is expected that biofouled materials could attract invertebrates capable of grazing on plastic inhabitants (Reisser et al. 2014), which increases the biofouling ratio at the same time (Lobelle and Cunliffe 2011). A consequence of biofouled material transmission is the transport of non-native or “alien” species. Microorganisms that naturally occur in one part of marine debris can be found in new distinct habitats, which could have a negative influence on marine ecosystems (De Tender et al. 2015; Debroas et al. 2017; Zettler et al. 2013). It still remains unknown how the transfer of non-native or invasive species into novel environments (Bryant et al. 2016; Maes et al. 2017) will cause change in the original ecosystems (De Tender et al. 2015). However, most of the interrelations between plastic waste and cold-marine habitats should be investigated more precisely.

## Plastic (bio)degradation process

Biodegradation is a process by which microbial organisms (mainly bacteria and fungi) transform or alter (through metabolic or enzymatic action) the structure of chemicals introduced into the environment (Muthu 2014). As noted previously, once plastic wastes enter the marine environment, the large particles of plastic are first fragmented to form microplastics or nanoplastic particles (Debroas et al. 2017; van Sebille et al. 2016). This multistage process is influenced by a variety of biotic and abiotic factors (Pauli et al. 2017). It means that microbial attachment on the surface and formation of biofilms depend not only on microorganisms’ abilities but also on the properties of the material and the surface structure (Donlan 2002) such as the surface roughness, topography, surface free energy, surface electrostatic interactions, and surface hydrophobicity (Rummel et al. 2017). Additionally, various factors related to environmental conditions such as salinity, temperature, oxygen level, and limitation of light have an impact on biofilm development (Dash et al. 2013; De Tender et al. 2015). Particularly, the increase in degradation rate by raising the temperature and humidity may be crucial. Different variations in sea temperature are expected to affect the rate of plastic degradation due to acceleration or inhibition of chemical reactions (O’Brine and Thompson 2010). Hence, the biomass of a fouling community influenced by different surface characteristics and environmental conditions (Pauli et al. 2017) is not always the same. It was suggested that bacterial adhesion to the plastic surface depends on the physicochemical surface and bacterial properties rather than on biological processes (Artham et al. 2009). At the same time, biotic and abiotic factors have an influence on released products (van Sebille et al. 2016).

Moreover, it was demonstrated that the weathering process is an important factor in degradation of plastic. The loss of physical integrity causes increasing availability of the surface for colonization by microorganisms (Rummel et al. 2017). Degradation mechanisms in the marine environment are not clear. The authors of the North Atlantic study observed microbial cells in pits on the plastic surface, which led them to implicate plastic-associated microbes in potential degradation of the plastic surface (Oberbeckmann et al. 2016). As mentioned above, the attachment is a key initiation process for degradation (Webb et al. 2009). However, it was noted that even though bacteria can easily colonize plastic, there is no evidence of potential degradation during early attachment (Lobelle and Cunliffe 2011). Nonetheless, O’Brine and Thompson observed biofilm appearance on the surface of four types of plastic: oxo-biodegradable *d2w* and EPI polyethylene bags, compostable BioBag bags and standard polyethylene bags. The biofilm formation occurred after 4 weeks of exposure in the shallow waters of the North Sea (O’Brine and Thompson 2010). Moreover, it was observed that the degradation of biodegradable bags was higher than polyethylene (PE) bags, with 100% degradation of the compostable material between 16 and 24 weeks. In turn, Eich et al. noted biofilm formation on the plastic bag surface after 15 days of exposure to the marine environment (Eich et al. 2015). The amount of biofilm increased significantly within 33 days on polyethylene and biodegradable plastic bags distributed to a shallow benthic and pelagic habitat. Due to differentiation between biofilm communities observed on both plastic types in different environments, the authors suggested that the early biofilm formation and composition are affected by the plastic type and habitat. Unfortunately, in their study, mechanical tests did not reveal a reduction in tensile properties of both plastic types within 1 month of exposure. However, scanning electron microscopy analysis revealed alterations in the surface of the biodegradable plastic. They noticed that the lack of clear changes in the properties of material could be caused by too short a period of carrying out the experiment. Lobelle and Cunliffe (2011) noted that biofilm formation may be visible within 1 week. The hydrophobic features of polyethylene plastic food bags submerged at the sea-end of Queen Anne’s Battery (UK) changed during an experiment lasting 3 weeks, but they did not observe polyethylene-degrading organisms. Additionally, their study shows that removing the visible biofilm from plastic reverses its physicochemical properties (Lobelle and Cunliffe 2011). In a recent study, biofilm formation on PS and PE was investigated (Oberbeckmann et al. 2017). It was found that already after 2 weeks of incubation in cold marine water (coastal Baltic Sea) microplastics were covered by assemblages, and bacteria from the genus *Erythrobacter* were found on the microplastics. Bacteria from this genus are known for their ability to utilize polycyclic aromatic hydrocarbons (PAHs). Thus, it was suggested that members of *Erythrobacter* might be able to degrade PAH associated with plastic (Oberbeckmann et al. 2017). Although the knowledge about microorganisms degrading plastic in cold habitats is poor, De Tender et al. (2015) investigated biofilm development on a plastic surface and suggested that factors modeling the biofilms may help to identify species potentially involved in biodegradation.

These observations highlight that the degradation process in the marine environment has not been studied sufficiently so far.

## Microorganisms isolated from cold marine environments with the ability to degrade plastic

So far, only a few studies have investigated the degradation of plastic in cold habitats. The current research is more focused on the interactions between marine ecosystems such as deep-sea environments and their microbial inhabitants (Sekiguchi et al. 2010) or the relations between marine microorganisms and plastic in general. Even though some microorganisms are capable of degrading plastic, usually biodegradation is recognized to be low (Debroas et al. 2017). However, some studies indicate the potential of isolated cold marine bacteria to degrade plastic (Table 1). Unfortunately, the main problem with this study is identification of the isolated microorganisms even if this activity is confirmed. The 16s rRNA sequences recovered in most studies reveal the presence of mainly unknown organisms only distantly related to known isolates (Ravenschlag et al. 1999). The research on degrading microorganisms is mainly focused on searching for them in deep-sea sediments where temperature decreases below 4 °C (in the case of 90% of the sea floor) (Ravenschlag et al. 1999; Russell 1990). Two types of PCL-degrading bacteria were isolated from deep seawater at 320 m depth in Toyama Bay. The isolated strains were identified as the *Pseudomonas* genus and were able to degrade PCL at 4 °C (Sekiguchi et al. 2009). Moreover, Sekiguchi et al. isolated bacteria belonging to the *Shewanella*, *Moritella*, *Psychrobacter*, and *Pseudomonas* genera from deep-sea sediment samples obtained from a depth of 5000–7000 m. Six isolated strains showed degrading abilities against a biodegradable polyester PCL. The authors also tested other biodegradable plastics such as PLA, PBSA, PBS, and polyhydroxybutyrate (PHB), but no activity was observed (Sekiguchi et al. 2010). However, in the next report, it was stated that PCL, PHB, and PBS fibers were degradable in deep seawaters despite low temperatures. Next, more five PCL-degrading strains were isolated from deep water (320–650 m depth), identified as bacteria from the genera *Pseudomonas*, *Alcanivorax*, and *Tenacibaculum.* Two of them, *Pseudomonas* spp. strains RCL01 and TCL04, were found to be adapted to conditions of low temperature (4 °C) and high hydrostatic pressure (Sekiguchi et al. 2011). Raghul et al. observed visible cracks and grooves on the surface of a polyvinyl alcohol-low linear density polyethylene (PVA-LLDPE) blend film after 15 weeks of incubation with a bacterial consortium consisting of *Vibrio alginolyticus* and *Vibrio parahaemolyticus* isolated from the benthic zones of different marine environments from a depth of 8 m (Raghul et al. 2014). In a recent study, bacterial and fungal strains from arctic regions with the ability to degrade bioplastic were isolated (Urbanek et al. 2017). In that study, the microbial activity against PLA, PCL, PBS, and PBSA was tested. The highest activity was observed for fungal strains identified as *Clonostachys rosea* and *Trichoderma* sp., and bacterial strains belonging to the *Pseudomonas* and *Rhodococcus* genera. PCL films were 53% degraded (*w*/*w*) during 30 days of incubation. Moreover, abundant growth on PLA films was observed, which might suggest the capacity for PLA degradation under certain conditions (Urbanek et al. 2017) (Table 2).

**Table 1:** Microorganisms isolated from cold environment with capability for degradation of plastic

Microorganism	Source	Plastic	References
*Shewanella*, *Moritella* sp., *Psychrobacter* sp., *Pseudomonas* sp.	Deep-sea sediment, the Kurile and Japan Trenches	PCL	Sekiguchi et al. (2010)
*Vibrio alginolyticus*, *Vibrio parahemolyticus*	Benthic zones of marine environments	PVA-LLDPE	Raghul et al. (2014)
*Pseudomonas* sp., *Clonostachys rosea*, *Trichoderma* sp., *Rhodococcus* sp.	The Arctic soil	PCL, commercial available bag based on potato and corn starch	Urbanek et al. (2017)
*Zalerion maritimum*	Marine environment	PE	Paco et al. (2017)
*Aspergillus versicolor*, *Aspergillus* sp.	Kovalam coast—off the Bay of Bengal, 500 m away from shore at the depth of 5 m	LDPE	Pramila and Vijaya Ramesh (2011)
*Pseudomonas* sp.	Deep seawater of Tottori Prefecture and offshore in Toyama bay	PCL	Sekiguchi et al. (2009)
*Pseudomonas* sp., *Alcanivorax* sp., *Tenacibaculum* sp.	Deep seawater	Monofilament fibers of PCL, PHB/V, PBS	Sekiguchi et al. (2011)

**Table 2:** Marine microorganisms isolated from the plastic surface

Microorganism	Source	Plastic	References
*Phormidium*, *Lewinella*	Microbial communities attached to PET drinking bottles submerged in the North Sea off the UK coast^a^	PET	Oberbeckmann et al. (2016)
*Phormidium* sp., *Rivularia*	Microplastic from the North Atlantic	PP, PE	Zettler et al. (2013)
*Stanieria*, *Pseudophormidium*	Microbial communities attached to PET drinking bottles submerged in the North Sea off the UK coast^a^	PET	Oberbeckmann et al. (2014)
*Pseudophormidium* sp., *Phormidium* sp.	Plastic particles harvested off the coasts of the UK, Germany, and Denmark	PP, PE	Oberbeckmann et al. (2014, 2016)
*Proteobacteria*, *Bacteroides*	Microplastic harvested off the Belgian part of the North Sea	Microplastic	De Tender et al. (2015)
*Arcobacter Colwellia* spp.	Coastal marine sediments within the Humber Estuary, UK	LDPE	Harrison et al. (2011)

Biodegration process not proven

^a^Experiment in vivo

## Marine bacteria as potential candidates for biodegradation of plastic wastes

Most of our planet is permanently cold (< 5 °C) and uninhabited by humans. The reason for this is that more than 70% of Earth is covered by seawater, mostly deep ocean, of which two thirds has a remarkably constant temperature of approximately 2 °C (Russell 1990). Nevertheless, bacteria can exist under these unfavorable conditions. Microbial communities resistant to such conditions may show many unique features. Among a number of microbial abilities in cold areas, the ability to decompose plastic is mentioned increasingly. It was assumed that the increasing amount of plastic waste leaking to the oceans may provide a new substratum for benthic organisms (Pauli et al. 2017). It was shown that in seawater, plastic releases dissolved organic carbon, stimulating the activity of heterotrophic microbes (Romera-Castillo et al., 2018). Adaptation to new sources of carbon can create new features of microorganisms, particularly in the production of cold-active enzymes. The uniquely cold-adapted enzymes of polar microorganisms provide numerous opportunities for biotechnological exploitation and give new insights into a wide range of applied issues such as plastic pollution (Rampelotto 2014). Currently, enzymes from psychrophilic microorganisms are raising interest for many industrial applications due to ongoing attempts to decrease energy demand (Yadav et al. 2017). Lower temperature needed for the growth at which enzymatic activity is maintained may be a huge advantage in the degradation process due to reduction of electric energy usage for heating. Thus, potentially microorganisms from cold habitats could be employed in open area landfill. Among the prominent microbial agents being used for biodegradation, species belonging to *Pseudomonas*, *Streptomyces*, *Corynebacterium*, *Arthrobacter*, *Micrococcus*, and *Rhodococcus* are mentioned most often (Pathak and Navneet 2017); the microorganisms have also been found in cold environments. Besides *Pseudomonas and Micrococcus*, bacterial isolates from *Polaromonas*, *Micrococcus*, *Subtercola*, *Agreia*, *Leifsonia*, *Cryobacterium*, and *Flavobacterium* were isolated from the cryoconite of three glaciers located in northwest Spitsbergen. Moreover, 12 of the isolated strains were able to produce lipase (Singh et al. 2014), an enzyme that hydrolyses ester bonds in lipids and in some polyesters (Jaeger et al., 1995). Extracellular lipase activity was also detected in microbial strains isolated from Arctic sea ice of the Canada Basin. Here, microorganisms were identified as belonging to the genera *Colwellia*, *Marinomonas*, *Pseudoalteromonas*, *Pseudomonas*, and *Shewanella*. Interestingly, the relative lipase activity was still detected at 0 °C in 20–40% and 10–30% of psychrophilic and psychrotolerant strains, respectively (Yu et al. 2009). The finding of cold-adapted bacterial strains with lipase activity is potentially important in view of the biodegradation process because several lipases hydrolyze polyesters such as PCL (Tokiwa and Calabia 2004; Pathak and Navneet 2017). Furthermore, it can be expected that other enzymes secreted by bacteria isolated from cold environments will show biodegradable activity. Potential enzymes that could be described as biodegradable include the lipases mentioned above, depolymerases (PHA depolymerases, PHB depolymerases, PLA depolymerases, PCL depolymerases), esterases, proteinases (proteinase K against PLA), cutinases, ureases, and dehydratases (Pathak and Navneet 2017). The biodegradability rate could be increased by supplementing polymers with additives which affect their thermal sensitivity and UV-absorbing capacity. Chemically sensitive polymers are more available for microbial attachment (Pathak and Navneet 2017). It was demonstrated that bacteria able to grow at − 1 °C release the greatest quantities of proteases (Huston et al. 2000), which proved the huge potential capacity for enzyme production by polar bacteria. Despite these facts, we still lack information about the possibility of biodegradation of petrochemical plastic such as PCV or PET. However, in a recent study, a new enzyme, PETase, produced by *Ideonella sakaiensis*, has been characterized (Austin et al., 2018). Thus, it clearly shows that we still lack full information about the microbial potential for plastic degradation.

## Conclusion

Floating plastic wastes have a negative influence on marine species and ecosystems. However, there is still a lack of precise knowledge about the quantity, sources, transport, accumulation, and role of plastics in the oceans. Fortunately, the scientific and public awareness of plastic as a global threat is rising. Numerous actions focus on tackling plastic accumulation by encouraging active involvement of consumers, producers, industry, and companies (Löhr et al. 2017). In 2016, for the first time, more plastic packaging waste was recycled than landfilled (region of European Union/Norway/Switzerland). Unfortunately, in many countries, landfill is still the first option of treatment for plastic waste (PlasticsEurope 2017). Thus, searching for new solutions is needed. Aside from the 3R strategy—reduce, reuse, and recycle plastic waste—which everyone is aware of, two more Rs should be considered: energy recovery and molecular redesign. Notably, the latter is seen as an emerging and very important element of the strategy (Thompson et al. 2009). Development of new bioplastic materials and their widespread application should help to reduce the impact of plastics on the environment. Typically, renewable raw materials instead of crude oil are used for their production, which saves valuable fossil resources and makes them more susceptible to waste management by composting or anaerobic digestion to reduce the input into the environment. The research applies to both BP and conventional plastic and is focused on the microbial activity associated with biofilm development on plastic surfaces in the marine environment. As the effects of biofouling communities are poorly understood, interactions between plastics and microorganisms urgently need to be studied. Arctic microorganisms may have unique potential due to the environmental conditions of polar oceans, which differ from other marine ecosystems. Bacteria from these regions respond quickly to changing environmental patterns. Thus, the growing amount of plastic waste might force microorganisms to adapt to new substrates. Despite this possibility, the future of our planet depends on us and on our responsibility for the plastic waste problem. Natural adaptation of microorganisms might take too much time, and consequently the littering of the natural environment may be irreversible.
